# Burden and quality of life of caregivers of individuals with stroke in the Western Amazon

**DOI:** 10.47626/1679-4435-2025-1379

**Published:** 2025-09-22

**Authors:** Evandro Piccinelli da Silva, Isabela Saura Sartoreto Mallagoli, Eloizi Cezar dos Santos Piccinelli, Maria Aline do Nascimento Oliveira, Kleyton Góes Passos, Angélica Gonçalves Silva Belasco

**Affiliations:** 1 Universidade Federal do Acre, Cruzeiro do Sul, AC, Brazil; 2 Universidade Federal de São Paulo, São Paulo, SP, Brazil; 3 Afya Faculdade de Ciências Médicas, Cruzeiro do Sul, AC, Brazil

**Keywords:** caregivers, caregiver burden, quality of life, stroke., cuidadores, sobrecarga do cuidador, qualidade de vida, acidente vascular cerebral.

## Abstract

**Introduction:**

Stroke causes functional limitations and requires care from informal caregivers, who often experience caregiver burden and compromised quality of life.

**Objectives:**

We aimed to assess the burden and quality of life of caregivers of patients with stroke and correlate burden with sociodemographic and clinical aspects, quality of life of both the caregivers and the patients, and the functional independence of the patients.

**Methods:**

This cross-sectional study included 136 caregivers and their patients from Cruzeiro do Sul, Acre, Brazil. A caregiver burden scale (Zarit Burden Interview), a quality of life instrument (36-item Short-Form Health Survey), and a functional independence questionnaire (Barthel Index) were used. In addition, sociodemographic and clinical data on caregivers were collected.

**Results:**

Caregiver burden was moderate-to-severe in 78.7% and severe in 39.7%. All domains of caregiver quality of life were compromised except functional capacity. According to the logistic regression, caregiver burden was most associated with home ownership and living with the patient (p = 0.0046), low caregiver scores in the physical (p = 0.0007) and mental health (p = 0.0009) domains, and moderate-to-severe patient dependence (p = 0.0040).

**Conclusions:**

Caregiver burden was associated with home ownership and living with the patient, low physical and mental health scores, and moderate-to-severe patient dependence.

## Abstract (pt)

**Introdução:**

O acidente vascular encefálico causa limitações funcionais e exige atenção de cuidadores informais que frequentemente enfrentam sobrecarga e comprometimento da qualidade de vida.

**Objetivos:**

Avaliar a sobrecarga e a qualidade de vida de cuidadores de pessoas com acidente vascular encefálico e correlacionar sobrecarga aos aspectos sociodemográficos e clínicos, à qualidade de vida de cuidadores e pacientes e à independência funcional dos pacientes.

**Métodos:**

Estudo transversal, com 136 cuidadores e respectivos pacientes, em Cruzeiro do Sul, Acre. Foram utilizados uma escala de sobrecarga (Zarit Burden Interview), um instrumento de qualidade de vida (Medical Outcomes Short-Form Health Survey-36), um questionário de independência funcional (Índice de Barthel) e um questionário de dados sociodemográficos e clínicos de cuidadores.

**Resultados:**

78,7% dos cuidadores apresentaram sobrecarga moderada a severa e 39,7%, intensa. A qualidade de vida dos cuidadores mostrou-se comprometida, exceto na capacidade funcional. A regressão logística revelou que a sobrecarga do cuidador foi respondida por: morar em casa própria com o paciente (p = 0,0046), baixos escores nos domínios aspectos físicos (p = 0,0007) e saúde mental dos cuidadores (p = 0,0009) e dependência moderada a intensa dos pacientes (p = 0,0040).

**Conclusões:**

A sobrecarga dos cuidadores foi respondida por: possuir casa própria e morar com o paciente, baixos escores nos domínios aspectos físicos e saúde mental e dependência moderada a intensa dos pacientes.

## INTRODUCTION

Among non-communicable diseases, stroke has one of the top three mortality rates worldwide.^1^ Stroke is defined as an interruption of blood supply to the brain, resulting in compromised circulation, regional damage, and the death of neural cells.^2^ Since the ability to perform activities of daily living (ADL), such as dressing, eating, using the toilet, bathing, and bowel and urinary retention may be limited in stroke survivors, specific support from a caregiver may be necessary.^3^

Because the term “caregiver” has been defined as all individuals who provide care for others, caregivers can be characterized in different ways depending on their social context. Formal or professional caregivers receive compensation for their work, while informal caregivers are defined as family members, friends, or neighbors who provide unpaid care and assistance to dependent individuals.^4^

After hospital discharge, the stroke’s effects are not limited to the patient, but abruptly extend to the family, especially close relatives, who often take on the role of informal caregiver.^5^ Informal caregivers begin their task without preparation and rarely receive specific training on care for patients with stroke, which can trigger feelings of unpreparedness, fear, insecurity, anxiety, and can result in compromised health and quality of life (QoL).^6^

According to the World Health Organization, QoL is defined as “the perception of one’s place in life in the context of the culture and value systems in which one lives and in relation to one’s goals, expectations, standards and concerns.”^7^

The diverse tasks of informal caregivers, combined with a lack of support, limited knowledge about care techniques, the patient’s functional dependence and chronic illness, high costs, and uncertainty about the future, can result in caregiver burden. Caregiving interferes with normal work activities, potentially resulting in job loss, reduced self-care and leisure time, and social isolation.^8^,^9^

A study on caregivers of stroke patients in Singapore found that informal caregivers had high care caregiver burden up to 1 year after the stroke event.^10^ Caregivers of individuals with a moderate degree of functional disability presented caregiver burden and reduced QoL.^11^ The changes and responsibilities imposed on informal caregivers mean that the deterioration of their health often goes unnoticed, making them “hidden” patients who do not always have the time or means to take care of their own health.^12^

Therefore, it is essential to investigate caregiver burden and associated factors among informal caregivers to help develop strategies to reduce this condition, improve their QoL and, consequently, the quality of care they provide.^13^,^14^

We assessed the caregiver burden level and QoL of informal caregivers of patients with stroke and correlated caregiver burden with sociodemographic and clinical variables, caregiver QoL, and the QoL and functional independence of their patients.

## METHODS

This study was approved by the Universidade Federal de São Paulo Research Ethics Committee (ruling 5,082,183) and followed National Health Council Resolution 466, which regulates the guidelines and standards for research involving human beings.

This analytical cross-sectional study was conducted in the city of Cruzeiro do Sul, Acre, from March 2022 to April 2023 and followed Strengthening the Reporting of Observational Studies in Epidemiology guidelines.^15^

We focused on the population of informal caregivers of patients with stroke treated in the city of Cruzeiro do Sul. The number of people affected by stroke was determined by the Municipal Health Department through the electronic primary health care system. For this study, 15 Family Health Strategy units were randomly selected from a total of 25 units. These units treated 464 patients with stroke, 208 of whom had caregivers.

Inclusion criteria for this study were patients, whose stroke occurred at least three months ago, and their caregivers (both at least 18 years of age). Patients with other neurological diseases, institutionalized patients, those belonging to indigenous communities, and those with professional caregivers were excluded.

The sample size was calculated based on the number of patients with stroke who had a caregiver and were registered in the Cruzeiro do Sul Family Health Unit information system in 2022. Based on a 5% margin of error of and 95% reliability level, a minimum of 136 patients with stroke and their caregivers were necessary.

Stroke patients were identified and located through contact with nurses and community health agent teams who had access to the public health information system and were familiar with the patients. Data were collected by the lead researcher and two undergraduate nursing students from the Federal University of Acre, who were trained and qualified to perform data collection.

Data were collected through a previously scheduled home visit by a community health agent, during which the patients and caregivers were invited to participate in the study after the research objectives, participant confidentiality, and right to withdraw were explained. All participants provided written informed consent. The interview was conducted in a private area of the home selected by the participants.

Sociodemographic, clinical, and QoL data (Medical Outcome Study 36-item Short-Form Health Survey)^16^ were collected from the patients and caregivers, in addition to data on caregiver burden (Zarit Burden Interview)^17^ and the patient’s independence in activities of daily living (Barthel Index).^18^

The sociodemographic and clinical data included age, sex, marital status, education, place of residence, housing, children, caregiver/patient relationship, job abandonment, current employment situation, time as an informal caregiver, daily activities as a caregiver, physical activity after becoming a caregiver, treatment seeking due to depressive symptoms after becoming a caregiver, and the caregiver/patient relationship preand post-stroke.

The 36-item Short-Form Health Survey, a generic QoL instrument that has translated and validated for use in Brazil, consists of eight domains (functional capacity, physical aspects, pain, general health status, vitality, social aspects, emotional aspects, and mental health), with scores for each domain ranging from 0 (worst state) to 100 (best state).^16^

The Zarit Burden Interview was used to assess caregiver burden level. This scale consists of 22 questions with a Likert-type response scale ranging from 0 to 4 points (never = 0, rarely = 1, sometimes = 2, very often = 3, always = 4). Total scores range from 0 to 88 points, with higher scores indicating a higher level of caregiver burden. Caregiver burden was classified as: none (0 to 20 points), mild-to-moderate (21 to 40 points), moderate-to-severe (41 to 60 points), or severe (61 to 88 points).^17^

The Barthel Index was used to assess the patients’ functional independence. This instrument analyzes 10 ADL domains: feeding, bathing, daily activities, dressing, bowel and urinary retention, toileting, chair-to-bed transfer, and mobility on flat surfaces and stairs. Total scores range from 0 to 100 points. We used the following cut-off points: total dependence (< 20 points), severe dependence (20-35 points), moderate dependence (40-55 points), mild dependence (60-95 points), and independence (> 95 points).^18^

Descriptive and inferential analyses of the sample were performed using IBM SPSS Statistics 23. The chi-square test as used to investigate the relationship between caregiver burden and sociodemographic and clinical characteristics, which was complemented by the likelihood ratio test when necessary. The Kruskal-Wallis test was used to analyze the association between caregiver burden and QoL for both the caregiver and the patient. We also compared the Barthel Index classification with the caregiver burden classification using the likelihood ratio test.

To identify factors that influenced caregiver burden, we applied logistic regression using a forward selection method. A 5% significance level was used in all statistical tests (p < 0.05).

## RESULTS

The sample consisted of 136 informal caregivers and their 136 patients. Most caregivers were women (82.4%), and their mean age was 51.1 years. Most were married or in a stable relationship (64.7%), had completed elementary school (52.2%), owned their own home (84.6%), lived in an urban area (85.3%), had children (87.5%), were unemployed (49.3%), had left their jobs because of caregiver responsibilities (43.4%), dedicated more than 8 hours a day to caregiver activities (79.4%), had cared for the patient for ≥ 3 years (61.8%), did not exercise after assuming the caregiver role (79.4%), and reported having sought health services due to depressive symptoms after becoming a caregiver (41.2%). The caregiver-patient relationship after the stroke was troubled, poor, or very poor in 19.9% of the cases (Table 1).

**Table 1:** Association between caregiver burden, sociodemographic and clinical characteristics, and relationship with the patient, March 2022 to April 2023, Cruzeiro do Sul, Acre, Brazil

Characteristics	n (%)	p-value
Sex		
Female	112 (82.4)	0.54^*^
Male	24 (17.6)	
Marital status		
Married/cohabiting	88 (64.7)	0.51†
Single	32 (23.5)	
Separated/divorced/widowed	16 (11.8)	
Education		
None	18 (13.2)	0.29†
Incomplete elementary school	33 (24.3)	
Complete elementary school	20 (14.7)	
Incomplete/complete high school	46 (33.8)	
Incomplete/complete higher education	19 (14.0)	
Area of residence		
Rural	20 (14.7)	0.05†
Urban	116 (85.3)	
Type of residence		
Homeowner	115 (84.6)	0.09†
Renting/living with family	21 (15.4)	
Children		
Yes	119 (87.5)	0.05†
No	17 (12.5)	
Degree of kinship with the patient		
Father/mother/brother	16 (11.8)	0.63†
Wife/husband/partner	42 (30.9)	
Child	56 (41.2)	
Other relative/friend	27 (19.9)	
Quit job to perform caregiving		
Yes	59 (43.4)	0.01^*^
No	77 (56.6)	
Current professional status		
Retired	41 (30.1)	0.31^*^
Employed	28 (20.6)	
Unemployed	67 (49.3)	
Time as informal caregiver (years)		
< 1	22 (16.2)	0.02†
> 1	19 (14.0)	
> 2	11 (8.1)	
≥ 3	84 (61.8)	
Daily work hours as a caregiver		
< 6	17 (12.5)	0.01†
6-8	11 (8.1)	
> 8	108 (79.4)	
Physical activity after becoming an informal caregiver		
Yes	28 (20.6)	0.004^*^
No	108 (79.4)	
Seeking health services due to symptoms of depression after becoming a caregiver		
Yes	56 (41.2)	0.0002^*^
No	80 (58.8)	
Pre-stroke caregiver/patient relationship		
Excellent	19 (14.0)	0.01†
Very good	23 (16.9)	
Good	75 (55.1)	
Troubled/poor/terrible	19 (14)	
Caregiver/patient relationship post-stroke		
Excellent	24 (17.6)	0.08^*^
Very good	34 (25.0)	
Good	51 (37.5)	
Troubled/poor/very poor	27 (19.9)	
Total	136 (100.0)	

% = Relative frequency; n = absolute frequency.

* Chi-square test.

† Likelihood ratio test.

Most of the 136 patients with stroke were male (55.1%), their mean age was 72.1 years, most were of mixed race (65.4%), most were married or in a stable relationship (50%) and most had suffered an ischemic stroke (77.2%).

As shown in Table 2, a significant proportion of the caregivers experienced caregiver burden, which was moderate-to-severe in 39.7%. Caregiver QoL was compromised in all domains except functional capacity (score = 72.1).

**Table 2:** Caregiver burden and QoL domains in informal caregivers and level of functional dependence and QoL domains in patients with stroke, March 2022 to April 2023, Cruzeiro do Sul, Acre, Brazil

Variable	Category	Mean (SD) or n (%)
Zarit Burden Interview (ZBI)		36.48 (17.7)
Classification of caregiver burden according to the ZBI score	No caregiver burden	29 (21.3%)
	Mild-to-moderate caregiver burden	53 (39.0%)
	Moderate-to-severe caregiver burden	38 (27.9%)
	Severe caregiver burden	16 (11.8%)
QoL (caregiver)	Functional capacity	72.1 (32.3)
	Physical aspects	49.4 (48.1)
	Pain	55.8 (29.7)
	General health	52.7 (26.4)
	Vitality	50.9 (26.7)
	Social aspects	60.9 (29.7)
	Emotional aspects	51.9 (48.8)
	Mental health	55.1 (25.4)
QoL (patient)	Functional capacity	16.4 (26.6)
	Physical aspects	16.5 (35.5)
	Pain	45.7 (26.4)
	General health	32.9 (24.1)
	Vitality	36.8 (24.4)
	Social aspects	34.8 (32.5)
	Emotional aspects	16.9 (36.7)
	Mental health	48.1 (24.0)
Barthel Index (patient)	Total/severe dependence	37 (27.2%)
	Moderate dependence	42 (30.9%)
	Mild dependence	47 (34.6%)
	Total independence	10 (7.4%)
Total		136 (100.0%)
		10 (7.4%)

% = relative frequency; stroke = cerebrovascular accident; SD = standard deviation; n = absolute frequency; QV = quality of life.

Most patients had some level of functional dependence in ADL: 34.6% had mild dependence, 30.9% had moderate dependence, and 27.2% had severe or total dependence. In patients with stroke, the scores for all QoL domains were < 50, with functional capacity (16.4), physical aspects (16.5), and emotional aspects (16.9) being the most affected.

As shown in Table 3, numerous significant correlations were found between caregiver burden and the QoL domains of both the caregiver and the patient. Moderate-to-severe and severe caregiver burden were correlated with moderate patient dependence. In the multiple logistic regression analysis (Table 4), the variables that best explained mild-to-moderate, moderate-to-severe, and severe caregiver burden were: home ownership, caring for a patient with a moderate degree of dependence, and low caregiver physical and mental health scores.

**Table 3:** Correlation between caregiver burden, quality of life domains of informal caregivers (136) and patients (136), and patient independence in activities of daily living, March 2022 to April 2023, Cruzeiro do Sul, Acre, Brazil

Caregiver burden (ZBI)	None	Mild-Mod	Mod-Sev	Sev	p-value
QoL domains (caregiver)^*^ mean (SD)					
Functional capacity	87.8 (22.4)	74.4 (30.2)	63.7 (33.9)	56.6 (39.3)	0.0078
Physical aspects	93.1 (25.8)	49.5 (46.4)	30.9 (45.6)	14.1 (34.1)	< 0.0001
Pain	80.8 (23.8)	57.3 (27.2)	42.8 (25.9)	37.1 (25.5)	< 0.0001
General health	67.9 (21.6)	59.7 (25.2)	41.5 (23.3)	28.8 (17.9)	< 0.0001
Vitality	74.3 (20.8)	52.5 (25.7)	40.4 (18.6)	28.7 (24.9)	< 0.0001
Social aspects	79.7 (26.6)	67.9 (25.8)	49.0 (26.1)	32.0 (24.1)	< 0.0001
Emotional aspects	89.7 (28.3)	55.9 (48.4)	31.6 (45.8)	18.7 (40.3)	< 0.0001
Mental health	75.2 (18.8)	59.5 (23.7)	45.0 (20.1)	28.7 (19.3)	< 0.0001
QoL domains (patient)^*^ mean (SD)					
Functional capacity	26.1 (32.3)	19.9 (29.9)	9.9 (17.6)	3.4 (8.9)	0.0092
Physical aspects	31.9 (42.7)	21.2 (39.9)	5.3 (22.6)	0 (0.0)	0.0008
Pain	51.3 (27.7)	48.3 (27.9)	40.4 (22.1)	40.0 (27.9)	0.1369
General health	46.3 (22.4)	36.5 (25.9)	24.7 (16.4)	16.6 (21.3)	< 0.0001
Vitality	51.9 (23.5)	42.7 (22.8)	24.1 (16.9)	20.3 (23.9)	< 0.0001
Social aspects	48.3 (38.6)	38.9 (32.4)	26.9 (25.9)	15.6 (21.6)	0.0068
Emotional aspects	36.8 (48.3)	18.8 (38.4)	6.1 (23.1)	0 (0.0)	0.0025
Mental health	58.9 (23.3)	54.5 (23.3)	39.3 (19.1)	28.7 (20.9)	< 0.0001
Barthel Index† n (%)					
Total/severe dependence	5 (17.2%)	16 (30.2%)	11 (28.9%)	5 (31.3%)	0.0013
Moderate dependence	3 (10.3%)	13 (24.5%)	19 (50.0%)	7 (43.8%)	
Mild dependence	17 (58.6%)	19 (35.8%)	8 (21.1%)	3 (18.8%)	
Total independence	4 (13.8%)	5 (9.4%)	0 (0.0%)	1 (6.3%)	

% = relative frequency; Mod = moderate; n = absolute frequency; QoL = quality of life; Sev = severe; SD = standard deviation; ZBI = Zarit Burden Interview.

* Kruskal-Wallis test.

† Likelihood ratio test.

**Table 4:** The set of variables that best explained caregiver burden in multiple logistic regression, March 2022 to April 2023, Cruzeiro do Sul, Acre, Brazil

	Estimate	p-value	OR	95%CI
Constant	4.22	0.0007	67.89	
Housing (living in own home vs. rented house or room/relative’s home)	2.43	0.0046	11.35	2.11-60.92
Barthel Index (total/severe dependence vs. mild dependence)	1.76	0.0233	5.84	1.27-26.79
Barthel Index (moderate dependence vs. mild dependence)	2.88	0.0040	17.87	2.51-127.02
Barthel Index (total independence vs. mild dependence)	-0.55	0.5995	0.58	0.07-4.51
Caregiver - physical aspects	-0.03	0.0007	0.97	0.95-0.99
Caregiver - mental health	-0.06	0.0009	0.94	0.91-0.98

OR = odds ratio.

The odds ratio for caregiver physical and mental health domain scores was < 1, that is, for each unit lower, caregiver burden increased.

## DISCUSSION

Traditionally, informal caregivers care for dependent relatives, based on family ties and geographic proximity.^11^,^12^ Our results, which were similar those of previous studies,^11^,^12^ indicated that most informal caregivers are children and spouses, predominantly female, with a mean age of 51.1 years, and a low education level.

Most informal caregivers lived in their own home with the stroke patient and had children, which may reinforce perceived caregiver burden. A similar study indicated that living with the patient significantly increases household chores and family responsibilities. Furthermore, caregiver burden is multifactorial and is influenced by variables such as the patient’s functional dependence level, stroke severity, and comorbidities.^10^

There was an association between caregiver burden, caregiver unemployment, and dedicating more than eight hours each day to caregiving. The extensive time spent providing care generally prevents caregivers from balancing caregiving with another job, resulting in quitting or being fired for repeated absences. Patients often become unemployed after a stroke or retire with a meager pension. Thus, the lack of funds to meet patient and caregiver needs contributes to caregiver burden. This scenario is in line with both national and international findings on the relationship between low income and caregiver burden.^10^,^19^,^20^

Stroke can cause temporary, reversible, and permanent consequences, requiring intensive daily care from caregivers.^15^ Similar to another study with the same type of population,^21^ most of our caregivers (61.8%) had been providing care the patient for more than 3 years, and most performed more than 8 hours of patient care each day.^9^,^10^,^20^ The long hours and prolonged duration of informal caregiving contribute to caregiver burden.^9^,^10^,^20^

A relationship has been identified among informal caregivers between lack of physical activity after assuming their role and increased caregiver burden. This was also identified in another study, which reported that caregiving is complex and leads to inactivity, since caregivers tend to prioritize the patient’s needs over their own, leaving them with no time for leisure activities or exercise. This exclusive focus on caregiving can increase caregivers’ emotional and financial pressure, affecting their overall well-being and contributing to caregiver burden.^22^

A significant portion of the caregivers in this study sought health services due to depressive symptoms that emerged after assuming their role, which could partially explain the increased caregiver burden. Studies have indicated that caring for a loved one after a stroke negatively affects the caregiver’s mental health, resulting in depressive symptoms.^5^,^13^ Another study found that depressive symptoms in caregivers can increase mortality risk in stroke survivors.^23^ It is essential to provide adequate support and resources to caregivers to protect their mental health and ensure the quality of care they provide.^24^

Although no significant link has been found between caregiver burden and the relationship with the patient after a stroke, we observed a substantial number of troubled, poor, or very poor relationships. The relationship between patients with stroke and family caregivers can be challenging; conflicts arise from a reluctance to ask for help or in decision-making, which breeds resentment. Promoting open dialogue about capabilities, needs, and expectations while balancing autonomy and safety strengthens empathy and collaboration and contributes to a healthy and positive relationship between both parties.^25^

Caregiver burden was observed in most cases (78.7%), primarily a mild-to-moderate level (39%), followed by a moderate-to-severe level (27.9%), which is in line with other studies.^13^,^24^,^26^ Stroke results in significant motor and neurological restrictions that affect ADL, the ability to return to work, and social interactions. The emotional after-effects of stroke can further increase the patient’s dependence on family members.^24^ This dependence leads to caregiver burden and emotional stress for caregivers, affecting their well-being and their family and social relationships.^11^

The QoL of this sample of informal caregivers was compromised in every domain except functional capacity. There was an association between caregiver burden and QoL domains, particularly physical and mental health, which, in the regression analysis, along with other variables, accounted for caregiver burden. Similar results have been observed in studies on caregivers of patients with stroke.^8^,^9^

Caregiving is physically demanding for caregivers, since motor impairment prevents the patient from performing ADL. Thus, caregivers must provide many services for the patient, including bathing, chair-to-bed transfer, wheelchair pushing, positioning in bed, feeding, housekeeping, transportation to medical and physical therapy appointments or other rehabilitationrelated activities, in addition to many other physical tasks.^27^

The mental demands on caregivers derive from multiple responsibilities, including patient stimulation, managing the home and finances, and providing emotional support for the patient at the expense of their own dreams, employment, and leisure activities. This can lead to frustration and can negatively affect mental health, increasing the perceived caregiver burden.^9^,^28^ These findings are consistent with other research suggesting that mental health impairment in caregivers is associated with emotional factors, such as stress, anxiety, and emotional burden, which play a significant role in people’s lives. Deteriorating mental health not only harms the QoL of caregivers but also overburdens them and compromises the quality of care they provide.^29^,^30^

In this study, all patient QoL domains except pain were associated with different levels of caregiver burden, but they alone did not account for the level. The patient’s degree of functional dependence (moderate, severe, or total) determined different levels of caregiver burden: the greater the patient’s dependence in ADL, the greater the caregiver burden. This is consistent with other studies that have attributed greater care demand to increased caregiver burden, including physical, emotional, and psychological stress.^5^,^12^

### STUDY LIMITATIONS

Due to the cross-sectional design of this study, causal relationships could not be determined among the variables. Longitudinal research is needed to further clarify these relationships.

## CONCLUSIONS

Most informal caregivers of patients with stroke experience significant caregiver burden and compromised QoL, except for functional capacity. The main variables that increased caregiver burden included living in the same house as the patient, high levels of patient dependence in ADL, and low caregiver scores in the physical and mental health domains. These findings highlight the need for interventions for both post-stroke patients and informal caregivers to reduce the negative effects of caregiver burden and ensure quality of care.
